# Spreading activation in emotional memory networks and the cumulative effects of somatic markers

**DOI:** 10.1007/s40708-016-0054-2

**Published:** 2016-07-15

**Authors:** Paul S. Foster, Tyler Hubbard, Ransom W. Campbell, Jonathan Poole, Michael Pridmore, Chris Bell, David W. Harrison

**Affiliations:** 1 Psychology Department, Middle Tennessee State University, 1500 Greenland Drive, Murfreesboro, TN USA; 2 University of Florida, Gainesville, USA; 3 Virginia Polytechnic Institute, Blacksburg, USA

**Keywords:** Spreading activation, Somatic markers, Skin conductance, Heart rate, Emotion, Semantic memory, Emotional memory, Episodic memory, Psychophysiology

## Abstract

The theory of spreading activation proposes that the activation of a semantic memory node may spread along bidirectional associative links to other related nodes. Although this theory was originally proposed to explain semantic memory networks, a similar process may be said to exist with episodic or emotional memory networks. The Somatic Marker hypothesis proposes that remembering an emotional memory activates the somatic sensations associated with the memory. An integration of these two models suggests that as spreading activation in emotional memory networks increases, a greater number of associated somatic markers would become activated. This process would then result in greater changes in physiological functioning. We sought to investigate this possibility by having subjects recall words associated with sad and happy memories, in addition to a neutral condition. The average ages of the memories and the number of word memories recalled were then correlated with measures of heart rate and skin conductance. The results indicated significant positive correlations between the number of happy word memories and heart rate (*r* = .384, *p* = .022) and between the average ages of the sad memories and skin conductance (*r* = .556, *p* = .001). Unexpectedly, a significant negative relationship was found between the number of happy word memories and skin conductance (*r* = −.373, *p* = .025). The results provide partial support for our hypothesis, indicating that increasing spreading activation in emotional memory networks activates an increasing number of somatic markers and this is then reflected in greater physiological activity at the time of recalling the memories.

The theory of spreading activation in semantic memory networks by Collins and Loftus [2] proposes that semantic concepts or memories (e.g., bear) are represented as nodes within a larger conceptual network (e.g., animals). The nodes in the conceptual network are interconnected through bidirectional associative links. Conceptual nodes within the same semantic category (bear – deer) have stronger associative links than nodes from different semantic categories (bear – wrench). Also, the strength of the associations between semantic nodes within a conceptual network varies, with some connections being very strong (e.g., dogs and cats) and other being relatively weaker (e.g., dogs and beavers). Activation of a node will spread along the bidirectional associative links to related nodes within the network. The strength of the associations between nodes, and hence the extent of the spread of activation, is related to and determined by the frequency of use of the associations between conceptual nodes, or production frequency norms. Associated concepts that are frequently accessed will then be stronger than those that are less frequently accessed or distantly activated within or between cerebral systems.

We have thought that a similar process of spreading activation may also exist within episodic memory networks and in particular emotional memory networks [12]. Essentially, as with semantic memory networks, activation of a given emotional memory will result in activation of the events, thoughts, and feelings associated with the specific memory as well as closely related emotional memories. Also, recollection of older memories may result in the concurrent and subsequent activation of more emotional memory nodes than newer memories since older memories are more likely to have been activated more frequently and hence have stronger connections with a greater number of other associated memories. Hence, recollection of older memories should be associated with greater spreading activation than newer memories. Meyer [24] proposed that older memories have a greater possibility of having been recalled in different motivation contexts. Sato [33] found that recollection of more remote memories was associated with the evocation of both temporally distant and proximal memories sharing the same lifetime, activities, location, or other distinctive features as compared to more recent memories that only evoked the same period or proximal memories more often.

A number of researchers have reported cerebral activation within the frontal and/or temporal lobes in response to recollection of emotional memories, including anger [35], happy [14, 21], and sad memories [29]. The neurophysiological effects of memory age have also been investigated. Maguire et al. [23] reported no hippocampal sensitivity to memory age. However, increased ventrolateral prefrontal activity was found during retrieval of recent autobiographical memories. Differential hippocampal activation between recent and remote memories has been reported by others [27, 30]. Further, positive correlations between the ages of angry memories and changes in low (13–21 Hz) and high (21–32 Hz) beta EEG amplitude in the right frontal and parietal lobes have been reported [10]. The increase in beta EEG amplitude with increasing memory age may reflect synchronization of underlying cortical cell assemblies as a greater number of associated memories are recalled and simultaneously activated during spreading activation.

The same regions of the brain involved in the ages of memories and recollecting emotional memories are also involved in physiological functioning. Research has supported both an inhibitory and an excitatory role of the prefrontal cortex in electrodermal activity [31, 34]. Raine and colleagues reported the involvement of the prefrontal and temporal regions as well as the pons in mediating skin conductance orienting [32]. The prefrontal regions also have an inhibitory influence on cardiovascular functioning, with stimulation resulting in bradycardia and depressor responses [15]. The temporal lobes, and in particular the insular cortices, are also known to be involved in modulating cardiovascular functioning [1, 28, 38]. Additionally, we have reported significant correlations between cardiovascular activity and EEG, both at rest [11, 9] and in response to recollection of angry memories [10]. Hence, the shared involvement of the frontal and temporal regions in both physiological activity and emotional memory recollection may provide part of the mechanism whereby emotional memories generate changes in indices of physiological activity.

The somatic marker hypothesis [3, 4] proposes that experiencing or recollecting an emotional memory activates the somatic markers associated with the memory, including the visceral sensations associated with the memory. Integrating the somatic marker hypothesis with the spreading activation model of Collins and Loftus [2], along with the aforementioned research regarding the neurophysiology of emotional memories and physiological functioning, suggests that as spreading activation increases through emotional memory networks, an increasing number of somatic markers will become activated. As a result, increasing spreading activation in emotional memory networks will be associated with increasing changes in electrodermal and cardiovascular functioning. Hence, older emotional memories, which are more likely to have been recalled a greater number of times and have more connections with other emotional memories, should be associated with greater changes in electrodermal and cardiovascular responses as compared to newer, more recent emotional memories. Partial support for this was found in a study that examined the relationship between changes in skin conductance and emotional memory age. Specifically, positive correlations were found between changes in skin conductance and the ages of angry and mirthful memories [12]. Cerebral laterality evidence further provides for a relative lateralization of sympathetic tone and negative emotion within right cerebral systems (e.g., [39–42].

The purpose of our original investigation was to determine whether emotional memory age represents a potential confound in research using memories to induce emotions [12]. The findings contradicted our original hypothesis and were interpreted through an integration of the aforementioned spreading activation and somatic marker models. The purpose of the present investigation was to replicate and extend our earlier findings and to use a method that would result in a greater variability in ages of memories and provide different indices of measuring spreading activation. We have used the words generated from the controlled oral word association test (COWAT) as a basis for measuring spreading activation (see Foster et al. [8]) and used a similar paradigm in this investigation. Specifically, we used an emotional version of the COWAT (eCOWAT), which requires individuals to generate words that have a specific emotional meaning for them and that are associated with specific memories. The number of emotional words generated and the average age of the associated memories for the words may then be used as a measure of spreading activation in emotional memory networks. Our hypothesis was that the number of emotional words generated and the average ages of the emotional words would be positively correlated with heart rate and skin conductance responses.

## Methods

### Participants

Our sample included 30 undergraduates (10 men and 20 women) with an age range of 18–42 years (*M* = 21.10, *SD* = 4.85). Most participants were right-handed, with two left-handed participants and one ambidextrous participant. Exclusionary criteria included having a history of significant head injury, current use of psychotropic medication, and current psychological problems.

## Apparatus

### Beck depression inventory – II (BDI-II)

The BDI-II is a 21-item self-report questionnaire used for measuring the severity of depression. The items of the BDI-II address problems related to numerous psychological, cognitive, and physiological symptoms. Each item is rated by the patient on a scale of 0–3, with a range of possible scores from 0 to 63.

### Emotional controlled oral word association test (eCOWAT)

The eCOWAT requires the individual to generate as many words as possible from a specified emotion (happy and sad) within a 60-second time limit. The words had to be personally meaningful for them and tied to specific emotional memories.

### BIOPAC MP150

Skin conductance and heart rate were recorded using the BIOPAC MP150 system (BIOPAC Systems, Inc., Goleta, CA). Skin conductance was measured using a GSR100C amplifier with silver/silver chloride electrodes filled with signa gel electrode gel. Heart rate was measured using a PPG100C amplifier and a photoplethysmograph transducer. A sampling rate of 200 Hz was used for both skin conductance and heart rate.

## Procedure

This study was approved by the Institutional Review Board of Middle Tennessee State University. After providing written informed consent, the participants were asked to complete the BDI-II and were then connected to the MP150. The photoplethysmograph transducer used to collect heart rate was connected to the ring finger of the nondominant hand. The silver/silver chloride electrodes used to measure skin conductance were attached to the distal phalanges of the index and middle fingers of the same hand. They were then asked to sit quietly and relax for 2 minutes. All participants then received a neutral emotional condition that required them to list items they had bought in a grocery store. They were specifically instructed not to simply list items that may be bought in a grocery store. Rather, they were asked to list items that they had actually purchased, i.e., items for which there were specific memories. The participants were given 60 s to recall as many grocery store items as possible. Following this neutral condition, they were given the two emotional word conditions, the eCOWAT. Specifically, they were provided with the following instructions:

I’m going to give you an emotion, the name of a specific emotion, and I want you to think of as many words as you can that are related to that emotion. I don’t want you to list words that society or people in general think are related to the emotion. What I want you to do is to list words that have a personal meaning for you, they must be associated with specific memories of yours. For example, if the emotion was anger you might list the words red, car, and scratch because those words are tied to a memory of a red car you once owned that was scratched in an accident that was not your fault. Do not provide a description of the memory, or a sentence. I just want you to list individual words that are personally emotional for you. You will have 60 s to generate as many emotional words as possible. Any questions?

After these instructions, the participants were given the first emotion and permitted 60 s to recall specific words. Afterwards, the participants were given the other target emotion and, again, permitted 60 s to recall specific words. The neutral condition was always administered first to control for contamination from the emotional conditions. The happy and sad emotion conditions were counterbalanced to control for potential carryover and sequence effects. After each condition, the participants were asked to provide a brief description of the memories from the words and asked to indicate the age of the memory associated with each word. The primary variables of interest included the number of words recalled and the average age for all of the words recalled in each of the separate conditions. The ages of the memories were converted to days for all subsequent analyses. A period of 2 minutes separated each of the three conditions. Heart rate and skin conductance were continuously recorded during the neutral, happy, and sad conditions and were averaged across the 60-second time allotted to recall the emotional words. These averaged heart rate (beats per minute or bpm) and skin conductance (micromhos or µmhos) values were then used as the basis for conducting subsequent correlations.

## Results

Initial analyses were conducted to determine if differences existed between the different conditions in the number of words produced, average memory age, heart rate, and skin conductance. The results indicated a significant difference, *t*(29) = 3.91, *p* = .001, in the number of words recalled between the neutral condition (*M* = 15.23, *SD* = 5.69) and the happy condition (*M* = 10.60, *SD* = 3.36). A significant difference, *t*(29) = 7.19, *p* < .001, was also found between the number of neutral words recalled and the number of words recalled in the sad condition (*M* = 7.57, *SD* = 2.69). Finally, the difference in the number of words recalled between the happy and sad conditions was also significant, *t*(29) = 5.09, *p* < .001. The same results were found when analyzing the average ages of the memories associated with the words. Specifically, a significant difference, *t*(29) = −3.13, *p* = .004, was found between the average ages of the neutral word memories (*M* = 40.59, *SD* = 70.07) and the average ages of the happy word memories (*M* = 335.51, *SD* = 532.12). A significant difference, *t*(29) = −6.35, *p* < .001, was also found between the average neutral memory word age and the average sad word memory age (*M* = 705.33, *SD* = 561.04). Finally, the difference in the average ages of the happy and sad word memories was also significant, *t*(29) = −3.19, *p* = .003. No differences in heart rate were noted between any of the three conditions. Regarding skin conductance, a significant difference, *t*(29) = −6.16, *p* < .001, was found between the neutral condition (*M* = 8.66, *SD* = 3.75) and the happy condition (*M* = 10.56, *SD* = 4.62). A significant difference, *t*(29) = −5.60, *p* < .001, was also found between the neutral condition and the sad condition (*M* = 10.19, *SD* = 4.55). No significant difference in skin conductance was found between the happy and sad conditions. Consult Table 1 for the means and standard deviations of all variables included.

**Table 1:** Means and standard deviation of the primary variables of interest

Word memory parameter						
	N words	H words	S words	N age	H age	S age
Mean	15.23	10.60	7.57	40.59	335.51	705.33
S.D.	5.69	3.36	2.69	70.07	532.12	561.04
Physiological parameter						
	N HR	H HR	S HR	N SCR	H SCR	NSCR
Mean	83.43	83.77	82.27	8.66	10.56	10.19
S.D.	14.50	13.04	12.88	3.75	4.62	4.55

N represents Neutral, H represents Happy, and S represents Sad. HR denotes heart rate in beats per minute and SCR represents skin conductance in μmhos

**Fig. 1:**
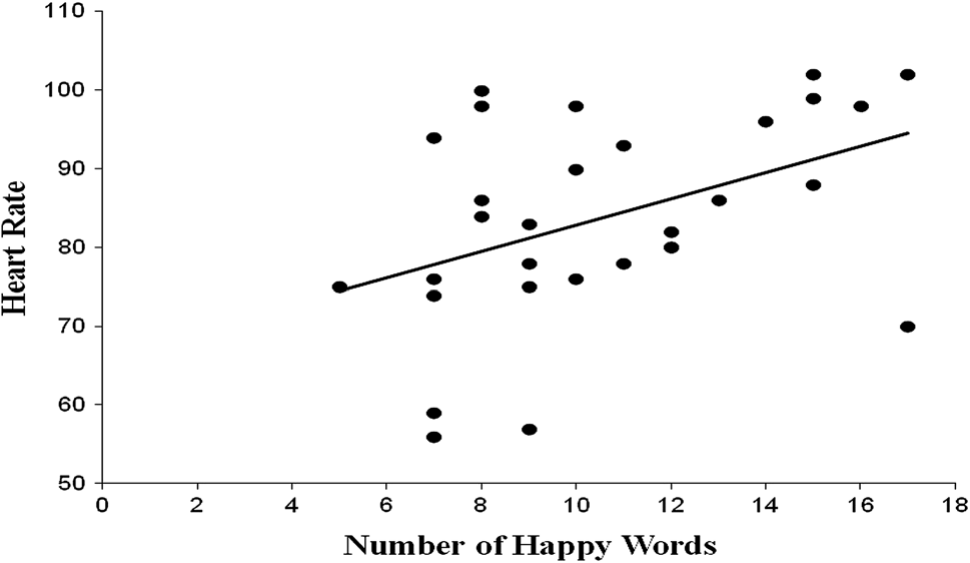
Relationship between the number of happy word memories recalled and heart rate (*r* = .384, *p* = .022)

**Fig. 2:**
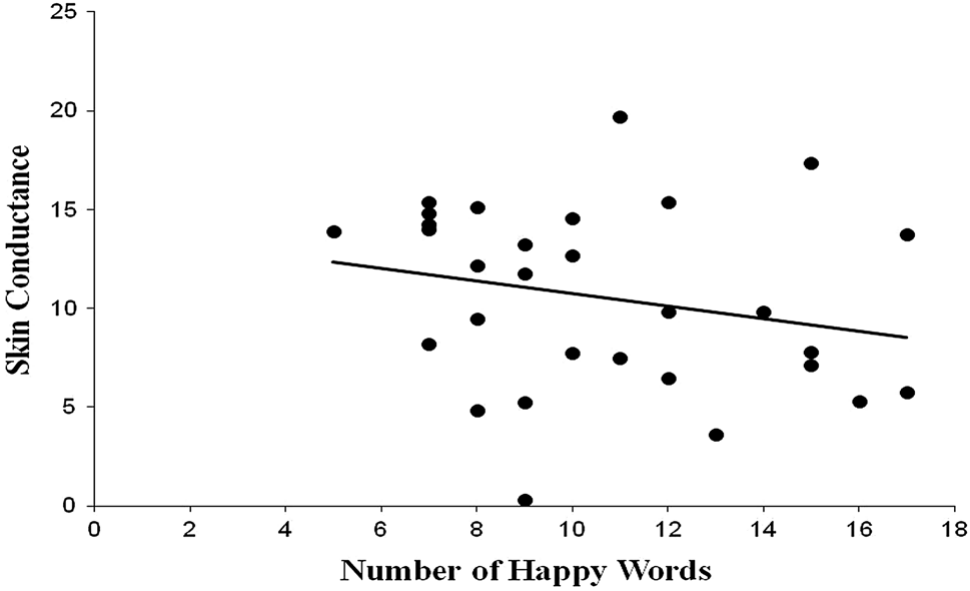
Relationship between the number of happy word memories recalled and skin conductance (*r* = −.373, *p* = .025)

**Fig. 3:**
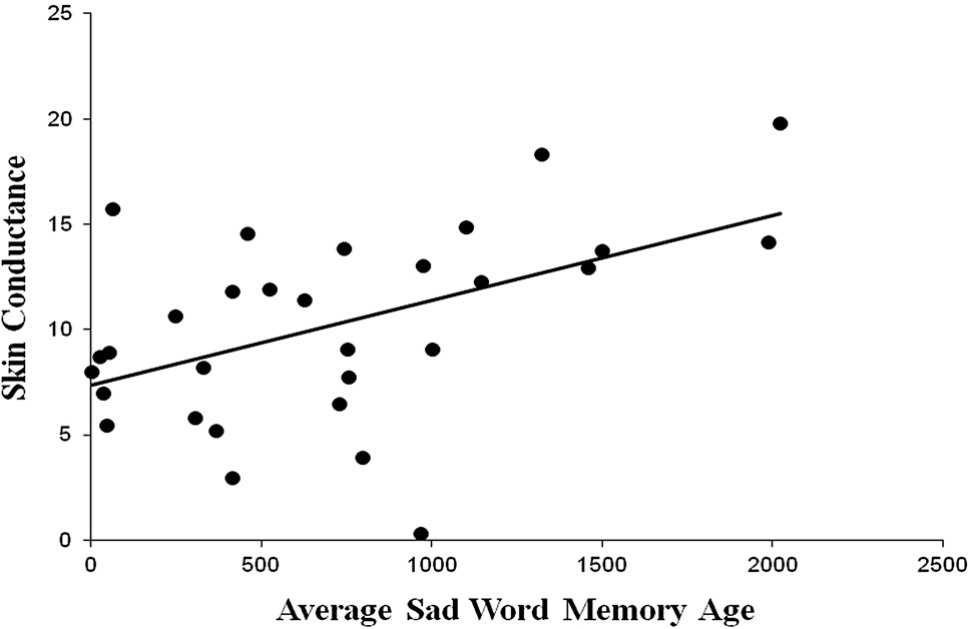
Relationship between the average ages of the sad memories and skin conductance (*r* = .556, *p* = .001)

Correlations were then conducted between each of the primary variables of interest. Regarding the number of words recalled, significant correlations were found between the number of happy word memories recalled and heart rate (*r* = .384, *p* = .022) and skin conductance (*r* = −.373, *p* = .025). No other significant correlations between the number of words recalled for each of the conditions and heart rate or skin conductance were found. Regarding the average ages of the word memories, a significant positive correlation was found between the average age of the sad word memories and skin conductance (*r* = .556, *p* = .001). No other significant correlations were found between the average ages of the word memories and heart rate or skin conductance (see Table 2). See Figs. 1, 2, 3 for scatterplots of significant correlations.

**Table 2:** Correlation coefficients for the primary variables of interest

	N words	H words	S words	N age	H age	S age
N HR	−.158 (.211)			−.155 (.215)		
H HR		.384 (.022)			.191 (.165)	
S HR			.288 (.069)			−.009 (.482)
N SCR	−.260 (.091)			−.241 (.109)		
H SCR		−.373 (.025)			.055 (.391)	
S SCR			−.014 (.473)			.556 (.001)

N represents Neutral, H represents Happy, and S represents Sad. HR denotes heart rate in beats per minute and SCR denotes skin conductance in μmhos. Probability is provided in parentheses

## Discussion

The results provide partial support for our hypothesis that increasing spreading activation in emotional memory networks would be associated with greater changes in heart rate and skin conductance due to the cumulative effect of an increasing number of somatic markers being activated. Both the number of emotional memory words and the average ages of the memories were significantly correlated with changes in heart rate and skin conductance. However, there were some differences between the happy and sad conditions as well as between the number of emotional memory words and the average ages of the memories. Specifically, there was a significant positive relationship between the number of happy word memories and heart rate but a significant negative relationship between the number of happy word memories and skin conductance. No significant relationships were found between the number of sad word memories and either heart rate or skin conductance. However, the average ages of the sad word memories was significantly positively correlated with skin conductance, but not with heart rate. Finally, the average ages of the happy word memories was not significantly correlated with either skin conductance or heart rate.

These findings support that spreading activation across emotional memory networks does seem to activate the somatic markers associated with these memories and these activations have a cumulative effect on current physiological functioning. However, in regard to heart rate, this effect seemed to exist only with the number of happy word memories. The reason that the same relationship was not found with the number of sad word memories may be because the sad condition was associated with the fewest number of memories recalled, which might also explain the lack of any significant association between the number of sad memories recalled and skin conductance. An unexpected finding was the negative relationship between the number of happy word memories and skin conductance. This suggests that the somatic markers regarding changes in skin conductance are associated with reduced electrodermal activity and hence the additive effect of these somatic markers would be to reduce electrodermal activity. The findings regarding happiness and skin conductance are rather mixed, some finding increases [22], others finding reductions [20], and still others finding no changes [13]. Hence, the overall effect of increasingly greater happy memories being recalled together with the somatic marker is to reduce skin conductance. The only significant relationship found with regard to the average ages of the emotional memories was with sadness and skin conductance. The reason for this finding might be that the sadness condition was associated with significantly older memories than the happy condition, and this would presumably indicate that as a result there were also more somatic markers activated. The findings are buttressed by the fact that the number of neutral word memories and the average age of the neutral memories were not significantly correlated with either heart rate or skin conductance, which would be expected given our hypothesis.

The number of words recalled differed reliably as a function of the affective valence associated with the words. Consistent with our theoretical underpinnings, access to words of one or of another valence may be restricted or promoted by proximity to functional systems, where associative strength would differ. Heightened fluency was found under the neutral affect condition with reliably few words produced under either happy or sad affective conditions in these comparisons. The least fluent condition was associated with negative affective word generation, where sad words were reliably less fluent than either happy or neutral words. This finding suggests that the production of emotional words requires more capacity and/or more distant associations from the neural systems needed for the production of neutral or happy word. Happy word fluency was significantly lower than that of neutral word fluency but significantly higher than that of sad word fluency, suggesting a moderating effect of positive affective systems on word production or fluency. Although positive affect expression has been related to left frontal processing (see Demaree, [5]; see also [16]) along with systems producing fluent speech output (for review see [17]), the anatomically distant and associatively far-field affective architecture within the right hemisphere ([19, 25, 26]; see also [18]) may contribute to the intermediate fluency effect.

This interpretation receives additional support in comparisons of either neutral or happy word production with that produced under the negative/sad condition, where the extent of lateralization and the requirement for potentially far-field associations with the right cerebral hemisphere would be increased. In this case, the far-field associations would lower word fluency as was found in the present study. Moreover, the demand for associative resources of the right hemisphere systems specialized for negative emotion (see would stress the capacity for regulatory control over sympathetic tone with heightened skin conductance, which was found under the affective fluency conditions. The present results provide only partial support for this with reliably elevated skin conductance under both sad and happy word generation conditions when compared with the neutral word fluency condition on the eCOWAT, whereas no significant difference was found in the comparison of skin conductance values between the happy and sad conditions.

Interestingly, the average age of the memory varied in a diametrically opposite direction to the number of words recalled across the three affect conditions. Neutral words reflected the most recent age for the memory of events, where the age of the memory increased with happy words and where the oldest or most persistent associations with memory events were found for the negative affect or sad condition. Cerebral laterality theory has long held that the linguistic processing systems within the left brain are specialized for rapid temporal sequencing and diminished persistence of the residual trace. This rapid sequential processing style has been related to the linguistic processing demands of the left cerebral hemisphere and where persistent memories of negative affective events may indeed have survival value, an attribute ascribed to the right cerebral hemisphere. Moreover, the present results are reminiscent of earlier discussions of the relative role of the right hemisphere in persistence, slow processing, and negative affect.

In the present investigation, sympathetic nervous system arousal was assessed using skin conductance measures and heart rate. Heightened sympathetic tone was found using skin conductance, where emotional word production (both for happy and for sad words) yielded reliably increased skin conductance over the neutral word generation condition. This relationship between the emotional associations and sympathetic tone yielded some interpretive consistency with the aforementioned relationships for persistence or age of the memory trace. More specifically, age of the memory was significantly related to the measurement of sympathetic tone with older memories predictive of not only affective valence (negative emotional words) but also increased sympathetic tone on measures of skin conductance.

In contrast to these comparisons providing partial support for the theoretical predictions, divergent findings resulted from the specific analyses of the number of happy words recalled as a function of heart rate as opposed to skin conductance measures. Earlier on, we provided evidence for the impact of happy word processing significantly lowering blood pressure and heart rate using the auditory affective verbal learning test (AAVLT; see [36, 37]). This instrument has been used not only for the investigation of learning within positive, negative, and neutral affective categories but also for the investigation of the impact of the learning material on autonomic nervous system functions, where blood pressure and heart rate may be directionally controlled as a function of the affective valence of the items learned. Simply, learning the positive list has been found to reliably lower blood pressure and heart rate, whereas learning the negative affective list reliably increases blood pressure and heart rate or sympathetic tone. Moreover, evaluation of hostile violent-prone individuals reveals a learning disability on acquisition of positive and neutral information, whereas no disability is present in the acquisition of negative affective information [5–7].

In the present study, the recollection and generation of happy words lowered skin conductance. Diametrically opposite results were found for measures of heart rate, where the production of more words was positively related to increased heart rate. Methodological differences between the present project and our earlier work may be partially responsible. Specifically, in the earlier manipulations cardiovascular measures (heart rate and blood pressure) were taken prior to and subsequent to the processing of the affective words. In the present study, the recording of heart rate was continuous and concurrent with the production of the happy words. Thus, the cardiovascular and cardiopulmonary demands associated with supporting active speech/word production may have impacted the heart rate measures in a divergent fashion with that of the skin conductance recordings.

There are some additional methodological limitations of our study that warrant mentioning. Although presentation of the happy and sad conditions was counterbalanced and two minutes separated these conditions, the possibility exists that the resulting arousal from one condition was not permitted enough time to return to baseline before administration of the following condition. Further, the questions regarding the ages of the memories and the descriptions of the memories were asked during the two minutes, which likely kept those memories activated during that time. This could also potentially explain the negative correlation for happiness. The age range of the subjects participating in the project might better be restricted by grouping factors in subsequent research as older subjects might well be expected to have older memories. The study was also correlational in nature and future researchers might choose to replicate these findings using a more empirically based approach. Finally, we did not collect a second baseline measure of physiological activity between the sad and happy memory conditions. As a result, we were not able to examine relative changes in heart rate and skin conductance as a function of emotional memory age or the number of emotional memories recalled. These limitations notwithstanding the results do provide support for the postulate that increasing spreading activation within emotional memory networks may also activate an increasing number of somatic markers, which lead to greater changes in heart rate and skin conductance.

## References

[1] Cechetto DF, Saper CB, Loewy AD, Spyer KM (1990). Role of the cerebral cortex in autonomic function. Central regulation of autonomic functions.

[2] Collins AM, Loftus EF (1975). A spreading-activation theory of semantic processing. Psychol Rev.

[3] Damasio AR (1994). Descartes’ error: Emotion, reason, and the human brain.

[4] Damasio AR (1995). On some functions of the human prefrontal cortex. Ann N Y Acad Sci.

[5] Everhart DE, Demaree HA, Harrison DW, Clark AV (2005). The merging of cognitive and affective neuroscience: studies of the affective auditory verbal learning test. Causes, role and influence of mood states.

[6] Everhart DE, Demaree HA, Harrison DW, Sher L (2008). Hostility and brain function: the impact of hostility on brain activity during affective verbal learning. Psychological factors and cardiovascular disorders: the role of psychiatric pathology and maladaptive personality features.

[7] Everhart DE, Demaree HA, Harrison DW (2008). The influence of hostility on electroencephalographic activity and memory functioning during an affective memory task. Clin Neurophysiol.

[8] Foster PS, Drago V, Ferguson B, Harrison DW (2008). Cerebral moderation of cardiovascular functioning: a functional cerebral systems perspective. Clin Neurophysiol.

[9] Foster PS, Drago V, FitzGerald DB, Skoblar BM, Crucian GP, Heilman KM (2008). Spreading activation of lexical-semantic networks in Parkinson’s disease. Neuropsychologia.

[10] Foster PS, Harrison DW (2004). Cerebral correlates of varying ages of emotional memories. Cogn Behav Neurology.

[11] Foster PS, Harrison DW (2006). Magnitude of cerebral asymmetry at rest: covariation with baseline cardiovascular activity. Brain Cogn.

[12] Foster PS, Webster DG (2001). Emotional memories: the relationship between age of memory and the corresponding changes in psychophysiological responses. Int J Psychophysiol.

[13] Foster PS, Webster DG, Smith EWL (1997). The psychophysiological differentiation of emotional memories. Imagin Cogn Personal.

[14] George MS, Ketter TA, Parekh PI, Horwitz B, Herscovitch P, Post RM (1995). Brain activity during transient sadness and happiness in healthy women. Am J Psychiatry.

[15] Hardy SGP, Holmes DE (1988). Prefrontal stimulus-produced hypotension in rat. Exp Brain Res.

[16] Harrison DW (2015). Positive and Negative Emotion. Brain Asymmetry and Neural Systems.

[17] Harrison DW (2015). Syndromes of the left brain. Brain asymmetry and neural systems.

[18] Harrison DW (2015). Positive and Negative Emotion. Brain asymmetry and neural systems.

[19] Heilman KM, Bowers D, Stein N, Leventhal B, Trabasso T (1990). Neuropsychological studies of emotional changes induced by right and left-hemisphere lesions. Psychological and biological approaches to emotion.

[20] Herridge ML, Harrison DW, Demaree HA (1997). Hostility, facial configuration, and bilateral asymmetry on galvanic skin response. Psychobiology.

[21] Lane RD, Reiman EM, Ahern GL, Schwartz GE, Davidson RJ (1997). Neuroanatomical correlates of happiness, sadness, and disgust. Am J Psychiatry.

[22] Levenson RW, Ekman P, Friesen WV (1990). Voluntary facial action generates emotion-specific autonomic nervous system activity. Psychophysiology.

[23] Maguire EA, Henson RNA, Mummery CJ, Frith CD (2001). Activity in prefrontal cortex, not hippocampus, varies parametrically with the increasing remoteness of memories. NeuroReport.

[24] Meyer DR (1984). The cerebral cortex: its roles in memory storage and remembering. Physiol Psychol.

[25] Mills CK (1912). The cerebral mechanisms of emotional expression. Trans Coll Physicians Phila.

[26] Mills CK (1912b) The cortical representation of emotion, with a discussion of some points in the general nervous mechanism of expression in its relation to organic nervous mental disease. In Proceedings of the american medico-psychological association 19: 297–300

[27] Niki K, Luo J (2002). An fMRI study on the time-limited role of the medial temporal lobe in long-term topographical autobiographic memory. J Cogn Neurosci.

[28] Oppenheimer SM, Kedem G, Martin WM (1996). Left-insular cortex lesions perturb cardiac autonomic tone in humans. Clin Auton Res.

[29] Partiot A, Grafman J, Sadato N, Wachs J, Hallett M (1995). Brain activation during the generation of non-emotional and emotional plans. NeuroReport.

[30] Piefke M, Weiss PH, Zilles K, Markowitsch HJ, Fink GR (2003). Differential remoteness and emotional tone modulate the neural correlates of autobiographical memory. Brain.

[31] Raine A, Lencz T, Roy JC, Bouscein W, Fowles DC, Gruzelier JH (1993). Brain imaging research on electrodermal activity in humans. Progress in electrodermal research.

[32] Raine A, Reynolds GP, Sheard C (1991). Neuroanatomical correlates of skin conductance orienting in normal humans: a magnetic resonance imaging study. Psychophysiology.

[33] Sato K (2002). Changes in the temporal organization of autobiographical memories. Psychol Rep.

[34] Sequeira H, Roy JC, Roy JC, Bouscein W, Fowles DC, Gruzelier JH (1993). Cortical and hypothalamic-limbic control of electrodermal responses. Progress in electrodermal research.

[35] Sidorova OA, Kostyunina MB, Kulikov MA (1992). Electroencephalographic and vegetative correlates of the mental reproduction of emotional states. Neurosci Behav Physiol.

[36] Snyder KA, Harrison DW (1997). The affective auditory verbal learning test. Arch Clin Neuropsychol.

[37] Snyder KA, Harrison DW, Shenal B (1997). The affective auditory verbal learning test: peripheral arousal correlates. Arch Clin Neuropsychol.

[38] Verberne AJM, Owens NC (1998). Cortical modulation of the cardiovascular system. Prog Neurobiol.

[39] Wittling W (1990). Psychophysiological correlates of human brain asymmetry: blood pressure changes during lateralized presentation of an emotionally laden film. Neuropsychologia.

[40] Wittling W, Davidson RJ, Hugdahl K (1995). Brain asymmetry in the control of autonomic-physiologic activity. Brain asymmetry.

[41] Wittling W, Block A, Genzel S, Schweiger E (1998). Hemisphere asymmetry in parasympathetic control of the heart. Neuropsychologia.

[42] Wittling W, Block A, Schweiger E, Genzel S (1998). Hemisphere asymmetry in sympathetic control of the human myocardium. Brain Cogn.

